# Duplex Collecting System With Ectopic Ureter Into the Posterior Urethra: A Case Report

**DOI:** 10.7759/cureus.23609

**Published:** 2022-03-29

**Authors:** Ali Al-Smair, Ahmad Saadeh, Omar Azizieh, Ahmad Al-Ali

**Affiliations:** 1 Department of Radiology, Medray International Radiology Center, Amman, JOR; 2 Faculty of Medicine, The University of Jordan, Amman, JOR; 3 Department of Radiology, Ministry of Health, Amman, JOR

**Keywords:** incontinence, case report, posterior urethra, ectopic ureter, duplex collecting system

## Abstract

The ureters are muscular tubes that carry urine from the kidneys to the urinary bladder and are typically implanted in the superolateral angle of the trigone of the urinary bladder. Although renal anomalies are common, especially in the kidneys, ectopia of the ureter is rare. Clinical presentation depends on the insertion of the ectopic ureter, varying from asymptomatic (mostly in males) to recurrent urinary tract infections (UTIs) and incontinence (mostly in females). Radiology is the best diagnostic tool to achieve a diagnosis. Ultrasound (US), intravenous renogram (IVR), micturition cystourethrogram (MCUG), and others are used to diagnose ectopic ureters, with US and MCUG being the gold standard modalities. Treatment depends on the functionality of the part drained by the ectopic ureter. Heminephroureterectomy and ureteroureterostomy are among surgical treatments for ectopic ureter, and it is either open or laparoscopic surgery. Herein, we present a case of a four-year-old female patient who presented with recurrent UTIs and incontinence.

## Introduction

The ureters are muscular tubes that carry urine from the kidneys to the urinary bladder. Embryologically, around the fourth week of development, an outpouching arises from the lower part of the mesonephric duct (Wolffian duct) called the ureteric bud. Eventually, the ureteric bud will become the future collecting system of the kidney including the ureter [1]. The duplex collecting system is the most common congenital renal tract abnormality, in which two ureteric buds arise from the mesonephric duct, or a single ureteric bud bifurcates. It can be detected antenatally by ultrasound, and most cases are asymptomatic [2]. However, it can present postnatally with a history of multiple urinary tract infections, urinary incontinence, abdominal pain, and even renal failure [3,4]. Many of the patient's symptoms depend on the insertion site of the ectopic ureter. Normally, ureters are inserted in the superolateral angle of the trigone of the urinary bladder. If any of the ureters was inserted away from its normal anatomical position, it is termed ectopic ureter [2]. In most cases, it is associated with duplex kidney, predominantly in females, in which the inferior ureteric bud is draining the upper renal pole and the superior ureteric bud is draining the lower renal pole [5]. The incidence of the ectopic ureter is rare, approximately 0.05%-0.025% [6]. Herein, we present a case of a four-year-old female patient who presented with a history of recurrent urinary tract infections and incontinence. This case emphasizes the importance of early detection of urological anomalies and radiological illustration of such cases.

## Case presentation

A four-year-old female patient presented to the clinic with a history of recurrent urinary tract infection (UTI) and urinary incontinence (UI). It occurred since birth; it does not increase with crying and standing; and she does not have the urge to void. She has a regular voiding habit apart from low-volume urine leakage, requiring changing pads multiple times daily. She was treated multiple times for UTIs, both upper and lower UTIs, and she had never been investigated for a cause. The patient was vitally stable and afebrile, and there were no signs of distress. She has normal looking external genitalia and urethral opening, no ectopic ureteral orifice, and no vaginal leak on physical examination.

**Figure 1:**
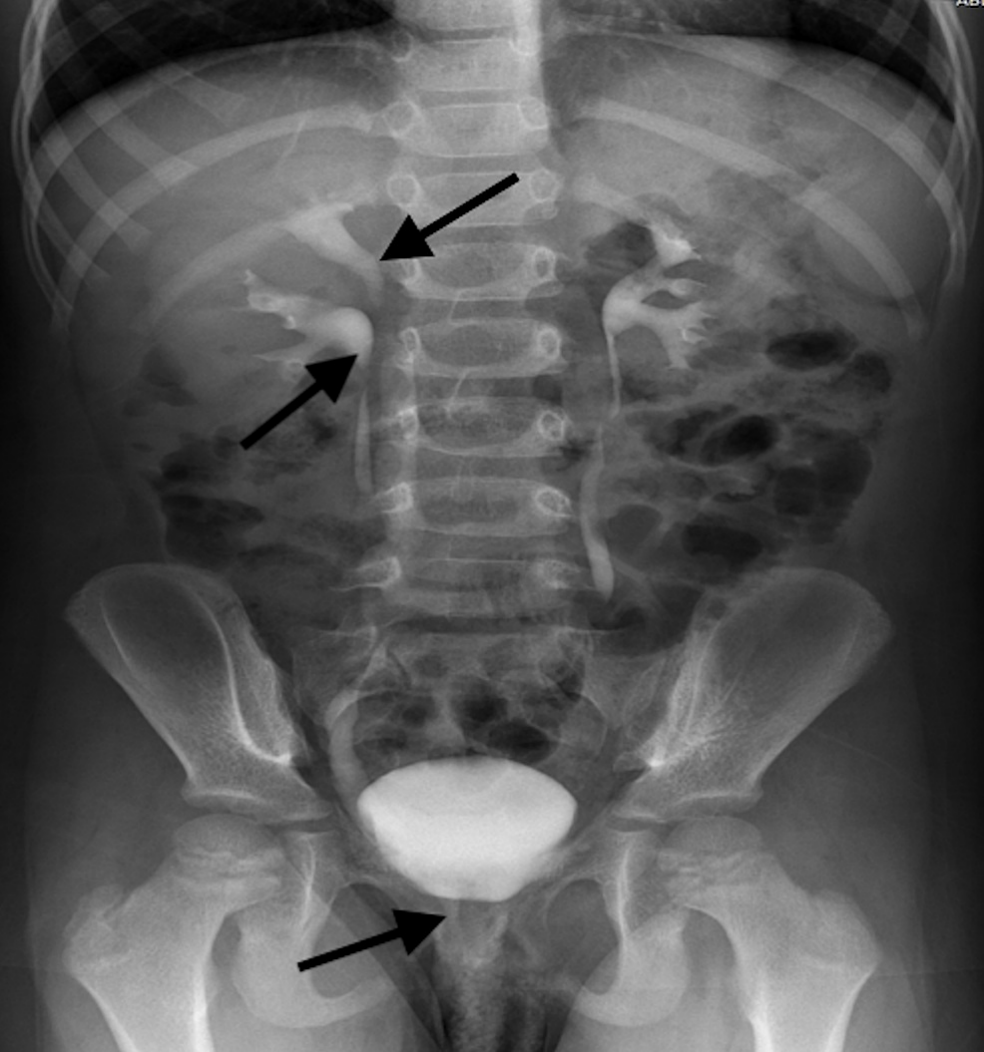
An IVU shows a right-sided complete double collecting system with ectopic insertion of the right upper moiety (black arrows). IVU: intravenous urogram.

Labs were sent, including complete blood count, creatinine, blood urea nitrogen (BUN), electrolytes, and urine analysis, all of which were normal. Abdominal ultrasound for both kidneys showed normal cortical differentiation, mild dilation of the right renal pelvis, and a duplex collecting system on the right side. The left side was completely normal. On the intravenous renogram (IVR), a right-sided duplex collecting system was noted with completely separated two ureters, and the upper ureter showed moderate dilation with abnormal insertion (Figure 1).

**Figure 2:**
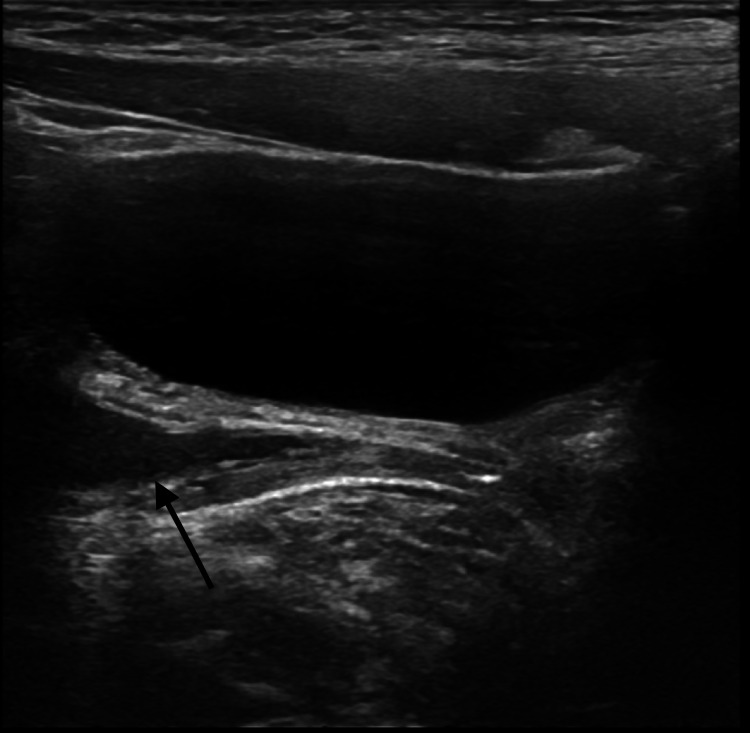
A US showing a well-defined elongated fluid-filled structure inserted into the proximal urethra (black arrow). US: ultrasound.

A complimentary ultrasound showed a well-defined elongated fluid-filled structure and ureteric insertion anterior to the vagina, suggesting the insertion of the posterior urethra (Figure 2).

**Figure 3:**
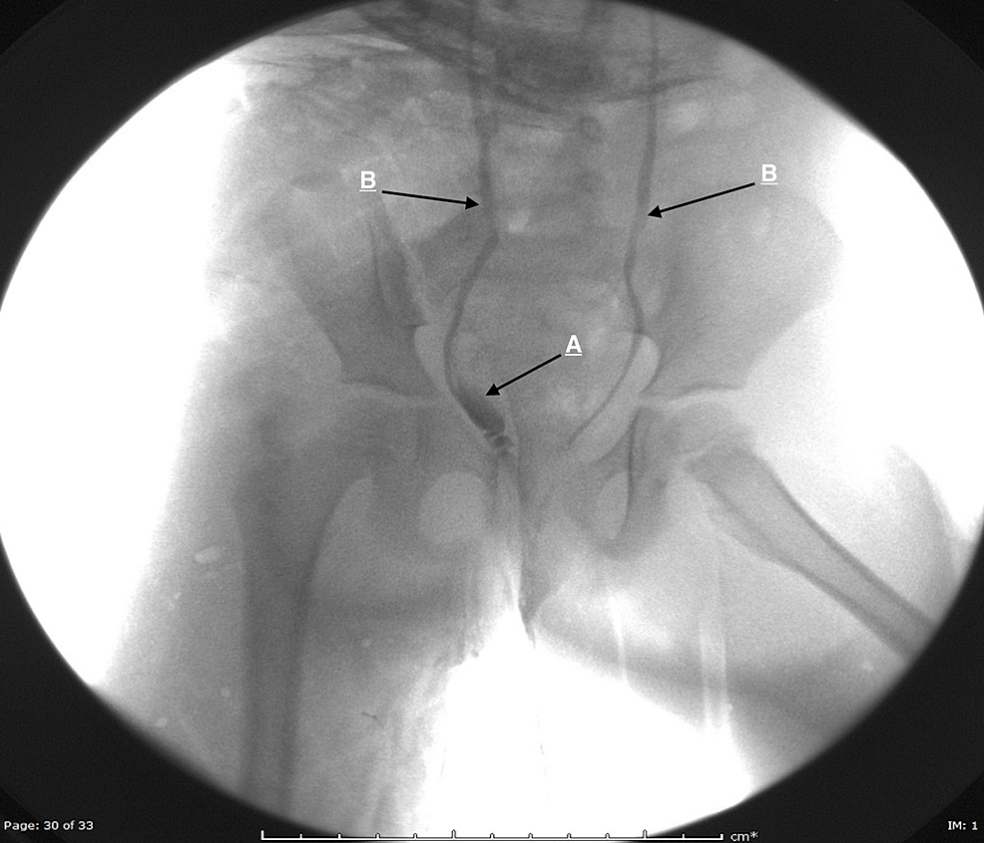
An MCUG image during voiding. A (black arrow) indicates reflux of the contrast from the urethra into the right ectopic ureter. B (black arrows) indicates bilateral reflux into the proper ureters. MCUG: micturition cystourethrogram.

The lower ureter has a normal diameter and insertion. The left kidney was expected with a normal single ureter. The urinary bladder was underfilled without focal lesion, stone, or ureterocele. On micturition cystourethrogram (MCUG), bilateral grade 2 vesicoureteral reflux was noted. During voiding, reflux of the contrast from the urethra to the lower third of the right ectopic ureter is noted, which appeared dilated, confirming the diagnosis of ectopic right upper moiety ureter insertion into the urethra (Figure 3).

Well-distended urinary bladder with a smooth outline, without significant postvoiding residual urine, was scanned. As aforementioned, the diagnosis of a right-sided duplex collecting system with an ectopic ureter was confirmed. The patient was started on nitrofurantoin prophylaxis and was referred to a pediatric urologist to discuss with the family the treatment options. A heminephroureterectomy was done, and the patient was doing well. She was prescribed nitrofurantoin prophylactically to prevent UTIs due to vesicoureteral reflux (VUR). Unfortunately, we lost the patient to follow-up.

## Discussion

When discussing congenital renal anomalies, the kidney is the most commonly involved organ of the urogenital system [6]. However, ectopia of the ureter is a rare anomaly with an incidence of 0.05%-0.025%, and the majority are females [7]. It is defined as a ureter that does not insert in its normal place.

Typically, the ureteric orifice opens into the trigone of the urinary bladder. The majority of ectopic ureter cases co-occur with the duplex collecting system, which frequently occurs with a dysplastic upper pole of the duplex system [5]. Duplex collecting system with ectopic ureter occurs in almost 30%, and the rest are single system with ectopic ureter as shown in the study by Choudhury et al. [3]. They also found isolated left-side involvement in almost 52%, bilaterally in 22%, and isolated right side in 26% [3]. In our case, we present a four-year-old female patient with a right duplex collecting system and two right completely separated ureters. The ectopic ureter was a continuation of the upper pole moiety.

Symptoms associated with ectopic ureter depend on the implantation site of the ureter. In males, the ectopic ureter commonly opens into the posterior urethral or the prostatic urethra, which occurs above the external sphincter and does not present with UI. In females, ectopias commonly open into the vulva and vagina, presenting a normal voiding pattern and UI [5,6]. Patients will also present with recurrent UTIs when the upper moiety fails to drain urine efficiently and with the copresence of vesicoureteral reflux. UTIs are important to be treated promptly to minimize renal scarring [8]. In our patient, she presented with recurrent UTIs, including pyelonephritis, two years ago, in which she required hospitalization.

The diagnosis of an ectopic ureter is mainly radiological, including ultrasound (US), IVR, MCUG, and others [9,10]. The US and MCUG are the standard imaging modalities used to diagnose ectopic ureter [9]. The US is the ideal initial modality used to detect urinary tract abnormalities, noninvasive, without radiation exposure, and provides a great anatomical image of the urinary tract. IVR is used to detect the ectopic ureter and its insertion point. However, sometimes it cannot be seen [10].

MCUG is the best modality for vesicoureteral reflux, which ideally should be performed after the first episode of UTI. It helps determine the grade and whether it occurs during filling or emptying the bladder [10]. The patient had an ultrasound a year or so in our case, which detected the double ureter. We reviewed the US and ordered an IVR, which showed a right-sided complete double collecting system. The complementary US showed a well-defined elongated fluid-filled structure inserted into the proximal urethra. For the recurrent history of UTIs, a voiding cystourethrogram (VCUG) was ordered to check for VUR. It showed reflux of contrast from the urethra into bilateral proper ureters and an ectopic ureter on the right side. The diagnosis of an ectopic right-sided ureter inserted in the proximal urethra posteriorly was confirmed, and bilateral VUR grade 2 was noted.

The main reason for surgical management of ectopic ureters is to preserve renal function [11], restore continence, and prevent recurrent UTIs. It depends on the part's functionality that is drained by the ectopic ureter. Dimercaptosuccinic acid (DMSA) scan is used to evaluate the functionality of the affected pole [7]. If it is nonfunctional, heminephroureterectomy can be performed. If it is functional, proximal and distal ureteroureterostomy procedures can be done [7,11]. In our case, the patient underwent a DMSA scan which showed a nonfunctional upper pole of the right kidney, probably due to recurrent UTIs. After discussing with the family, a heminephroureterectomy surgery was chosen. The patient underwent surgery with an uneventful postoperative period, and she was doing well. She was started on a prophylactic dose of nitrofurantoin to prevent UTIs due to the VUR. Unfortunately, we lost the patient to follow-up. 

## Conclusions

Ureteric ectopia is a rare abnormality, and it is usually associated with a duplex collecting system. In most cases, it is asymptomatic in males. However, it presents with incontinence in females. It is also associated with VUR and UTIs, which can lead to renal scarring and loss of function. Early diagnosis and treatment of such abnormalities are crucial to preserve renal function and maintain continence. Raising awareness about this abnormality, the best way to diagnose (using US, IVR, and MCUG images), and treatment will aid in better outcomes in those patients.

## References

[1] Rasouly HM, Lu W (2013). Lower urinary tract development and disease. Wiley Interdiscip Rev Syst Biol Med.

[2] Abyaksa R, Pramod SV, Siregar S (2021). Uterus as one of the ectopic ureter openings: case report. Urol Case Rep.

[3] Roy Choudhury S, Chadha R, Bagga D, Puri A, Debnath PR (2008). Spectrum of ectopic ureters in children. Pediatr Surg Int.

[4] Mathews R, Jeffs RD, Maizels M, Palmer LS, Docimo SG (1999). Single system ureteral ectopia in boys associated with bladder outlet obstruction. J Urol.

[5] Duicu C, Kiss E, Simu I, Aldea C (2018). A rare case of double-system with ectopic ureteral openings into vagina. Front Pediatr.

[6] Mikuz G (2019). Ectopias of the kidney, urinary tract organs, and male genitalia. Der Pathologe.

[7] Demirtas T, Tombul ST, Golbasi A, Sonmez G, Demirtas A (2021). The ectopic ureter opening into the vulva, which is a rare cause of lifelong urinary incontinence: treatment with ureteroureterostomy. Urol Case Rep.

[8] Michaud JE, Akhavan A (2017). Upper pole heminephrectomy versus lower pole ureteroureterostomy for ectopic upper pole ureters. Curr Urol Rep.

[9] Arevalo MK, Prieto JC, Cost N, Nuss G, Brown BJ, Baker LA (2017). Utility of retrograde ureterocelogram in management of complex ureterocele. J Pediatr Urol.

[10] Berrocal T, López-Pereira P, Arjonilla A, Gutiérrez J (2002). Anomalies of the distal ureter, bladder, and urethra in children: embryologic, radiologic, and pathologic features. Radiographics.

[11] Kim HH, Kang J, Kwak C, Byun SS, Oh SJ, Choi H (2004). Laparoscopy for definite localization and simultaneous treatment of ectopic ureter draining a dysplastic kidney in children. J Endourol.

